# Molecular Characteristics and Antioxidant Activity of Spruce (*Picea abies*) Hemicelluloses Isolated by Catalytic Oxidative Delignification

**DOI:** 10.3390/molecules27010266

**Published:** 2022-01-02

**Authors:** Valentina S. Borovkova, Yuriy N. Malyar, Irina G. Sudakova, Anna I. Chudina, Andrey M. Skripnikov, Olga Yu. Fetisova, Alexander S. Kazachenko, Angelina V. Miroshnikova, Dmitriy V. Zimonin, Vladislav A. Ionin, Anastasia A. Seliverstova, Ekaterina D. Samoylova, Noureddine Issaoui

**Affiliations:** 1 School of Non-Ferrous Metals and Materials Science, Siberian Federal University, pr. Svobodny 79, 660041 Krasnoyarsk, Russia; bing0015@mail.ru (V.S.B.); skripnikov@yandex.ru (A.M.S.); leo_lion_leo@mail.ru (A.S.K.); miroshnikova35@gmail.com (A.V.M.); zimonind89@mail.ru (D.V.Z.); sl79490@yandex.ru (V.A.I.); aaseliverstova@sfu-kras.ru (A.A.S.); yekaterina.sam09041998@mail.ru (E.D.S.); 2 Krasnoyarsk Science Center, Institute of Chemistry and Chemical Technology, Siberian Branch, Russian Academy of Sciences, Akademgorodok 50/24, 660036 Krasnoyarsk, Russia; sudakova_irina@mail.ru (I.G.S.); bai77@list.ru (A.I.C.); fou1978@mail.ru (O.Y.F.); 3 Laboratory of Quantum and Statistical Physics (LR18ES18), Faculty of Sciences, University of Monastir, Monastir 5079, Tunisia; issaoui_noureddine@yahoo.fr

**Keywords:** wood polysaccharides, delignification, hemicellulose, antioxidant activity, molecular weight distribution, gel permeation chromatography

## Abstract

Spruce (*Picea* *abies*) wood hemicelluloses have been obtained by the noncatalytic and catalytic oxidative delignification in the acetic acid-water-hydrogen peroxide medium in a processing time of 3–4 h and temperatures of 90–100 °C. In the catalytic process, the H_2_SO_4_, MnSO_4_, TiO_2_, and (NH_4_)_6_Mo_7_O_24_ catalysts have been used. A polysaccharide yield of up to 11.7 wt% has been found. The hemicellulose composition and structure have been studied by a complex of physicochemical methods, including gas and gel permeation chromatography, Fourier-transform infrared spectroscopy, and thermogravimetric analysis. The galactose:mannose:glucose:arabinose:xylose monomeric units in a ratio of 5:3:2:1:1 have been identified in the hemicelluloses by gas chromatography. Using gel permeation chromatography, the weight average molar mass M_w_ of hemicelluloses has been found to attain 47,654 g/mol in noncatalytic delignification and up to 42,793 g/mol in catalytic delignification. Based on the same technique, a method for determining the α and *k* parameters of the Mark–Kuhn–Houwink equation for hemicelluloses has been developed; it has been established that these parameters change between 0.33–1.01 and 1.57–472.17, respectively, depending on the catalyst concentration and process temperature and time. Moreover, the FTIR spectra of the hemicellulose samples contain all the bands characteristic of heteropolysaccharides, specifically, 1069 cm^−1^ (C–O–C and C–O–H), 1738 cm^−1^ (ester C=O), 1375 cm^−1^ (–C–CH_3_), 1243 cm^−1^ (–C–O–), etc. It has been determined by the thermogravimetric analysis that the hemicelluloses isolated from spruce wood are resistant to heating to temperatures of up to ~100 °C and, upon further heating, start destructing at an increasing rate. The antioxidant activity of the hemicelluloses has been examined using the compounds simulating the 2,2-diphenyl-2-picrylhydrazyl free radicals.

## 1. Introduction

Hemicelluloses (HCs) are plant cell wall polysaccharides, which are the second most widely used renewable plant polymers after cellulose in lignocellulosic materials. They represent heteropolysaccharides with a disordered polymer structure consisting of different sugar units [1], depending on a plant type and extraction method. The HCs that are dominant in softwood are partially acetylated galactoglucomannans and glucomannans (up to 20–25% of the wood weight) and arabinoglucuronoxylans (7–10% of the wood weight) [2,3].

Wood HCs are an abundant, but still unused by-products of the pulp and paper and hydrolysis industries. However, the early investigations charted the trends in using HCs in different production areas.

According to the literature data on the isolation and application of HCs, these natural polymers can be used in biomedicine owing to their nontoxicity, biocompatibility, and biodegradability [4,5]. It was proposed to employ HCs in packaging films [6] and 3D printing [7]. Deloule et al. [5], Gautam et al. [8], and Mikkonen et al. [9] demonstrated the wide possibilities of using the HCs extracted from conifers as prebiotics, adsorbents, and stabilizers of alkyd paints.

In the recent works devoted to the extraction of HCs from wood raw materials, methods for the HC isolation via the hot water extraction [10,11], pretreatment with hot water under pressure [12], alkaline treatment with hydrogen peroxide [13], etc. [14,15,16] were proposed. At present, new methods for HC production are being developed, which are based on using organic solvents, for example, carboxylic acids, as a delignification medium for wood processing. Among these promising methods is oxidative delignification in acetic acid-water-hydrogen peroxide medium in the presence of different catalysts [17,18,19]. The pretreatment using acetic acid with hydrogen peroxide allows the efficient fractionation of lignocellulosic materials and cleavage of the lignin aromatic rings at the preserved polymer structure of other carbohydrates [20,21]. Importantly, the delignification products consisting of soluble HCs and lignin depolymerization products are free of toxic chlorine and sulfur-containing compounds [1,22]. In addition, the presence of various catalysts, especially those based on variable valence metals, intensifies the delignification process due to the formation of peroxocomplexes, which are strong oxidants and destruct lignin more completely [23,24].

Norway spruce (*Picea abies*) is an evergreen coniferous widespread in most of Europe. Numerous studies showed that the components of the spruce wood and needles exhibit wide-range biological activities, including antioxidant [25], immunomodulatory [26], anti-cancer [27], anti-inflammatory, antifungal, and others [28,29].

This work is aimed at the extraction of HCs from *Picea abies* wood by oxidative delignification in acetic acid-water-hydrogen peroxide medium with the (NH4)_6_Mo_7_O_24_, MnSO_4_, TiO_2_, and H_2_SO_4_ catalysts and without them; gel permeation chromatography (GPC) examination of the dependence of the HC molar mass on the delignification conditions; gas chromatography (GC) investigations of the HC monosaccharide composition; analysis of the structural changes and thermal stability of the obtained samples by Fourier-transform infrared (FTIR) spectroscopy and thermogravimetric analysis (TGA); and study of the bioactivity of the HCs using the compound modeling free and hydroxyl radicals for determining their characteristics in terms of future application.

## 2. Results and Discussion

### 2.1. Delignification and Yield of the Hemicelluloses

The HCs of spruce wood were extracted from liquid products of oxidative delignification in the hydrogen peroxide-acetic acid-water medium after isolation of cellulose. The delignification conditions were chosen based on study [17], where it was shown that, at process temperatures of 90–100 °C, process times of 3–4 h, a hydrogen peroxide content of 6 wt%, an acetic acid content of 30 wt%, and a liquid/wood ratio (LWR) of 15, the deepest delignification of wood occurs, which ensures a residual lignin content below 1% in the cellulosic product. The dissolved HCs were isolated from the liquid delignification products by ethanol precipitation. 

The yields of HCs extracted from the products of the oxidative catalytic and noncatalytic delignification of spruce wood in the hydrogen peroxide-acetic acid-water medium are given in Table 1.

According to the data given in Table 1, the HC yield at a process temperature of 90 °C is mainly lower than that obtained at 100 °C. This is due to the fact that, at 100 °C, wood delignification is probably completed.

The use of the (NH_4_)_6_Mo_7_O_24_ and MnSO_4_ catalysts ensures a fairly high HC yield at 90 °C, while the HC yield obtained with the MnSO_4_ catalyst at 100 °C for 4 h decreases. The TiO_2_ catalyst works much more efficiently at process parameters of 100 °C and 3 h than at 100 °C and 4 h. When the H_2_SO_4_ catalyst is used, the HC yield is the lowest, which is apparently related to the strong destructive effect of this catalyst on polysaccharides. 

### 2.2. Monosaccharide Composition of the Hemicelluloses

**Figure 1:**
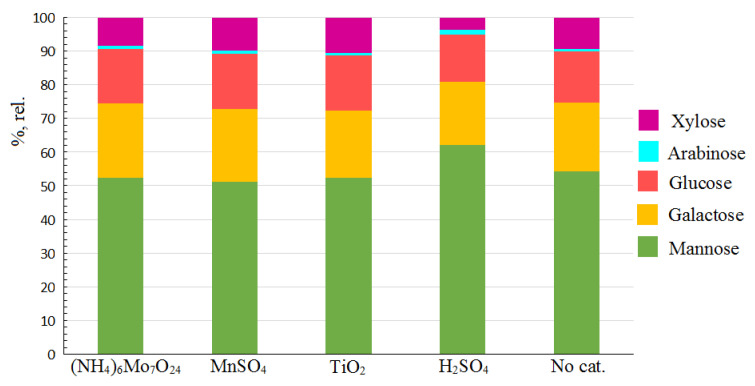
Monosaccharide composition of the hemicelluloses isolated from liquid products of oxidative delignification of spruce wood with different catalysts and without them (percentage of the total monosaccharide content).

The carbohydrate composition of the HCs obtained by the oxidative catalytic and noncatalytic delignification of spruce wood is shown in Figure 1. The HC monosaccharide composition revealed the presence of large amounts of mannose, galactose, and glucose and minor amounts of arabinose and xylose in a ratio of 5:3:2:1:1. The highest galactose and mannose contents in the HC composition point out that the dominant polysaccharide in the obtained HCs is galactoglucomannan. The presence of small amounts of xylose and arabinose is indicative of the presence of another important polysaccharide: arabinoglucuronoxylan. These components determined for the spruce (*Picea abies*) HCs are analogous to those reported in the previous studies on the HC isolation from coniferous, although their ratios are somewhat different [1,13]. 

### 2.3. Gel Permeation Chromatography Study of the Hemicelluloses

The determination of the conformational and molecular mass characteristics of the HCs obtained by oxidative delignification can open up new prospects for studying the interplay between the structure and properties of polysaccharides, which will make it possible to obtain HCs with controlled characteristics for further use in pharmacology, the food industry, etc. The information about the structure of the polysaccharide chain in an aqueous solution can be obtained by comparing molar masses with some hydrodynamic parameters, e.g., the intrinsic viscosity. This dependence is described by the Mark-Houwink-Sakurada (MHS) equation [η] = KM^α^. In the logarithmic coordinates, α is the slope of a straight, which characterizes the conformation of molecules in a solvent. The coefficient α is ∼0.0 for hard spheres, ∼0.5 for linear random coils in theta solvents (e.g., DMSO), 0.5–0.8 for linear polymers in thermodynamically good solvents, and 1.8–2.0 for rigid rods. The change in the coefficient K at the constant α values allows one to judge the branching of polymer molecules. A slope of ∼0.58 is typical of linear polymers in thermodynamically good solvents, while its lower values are indicative of branching (0.33 for compact spheres) [30].

**Figure 2:**
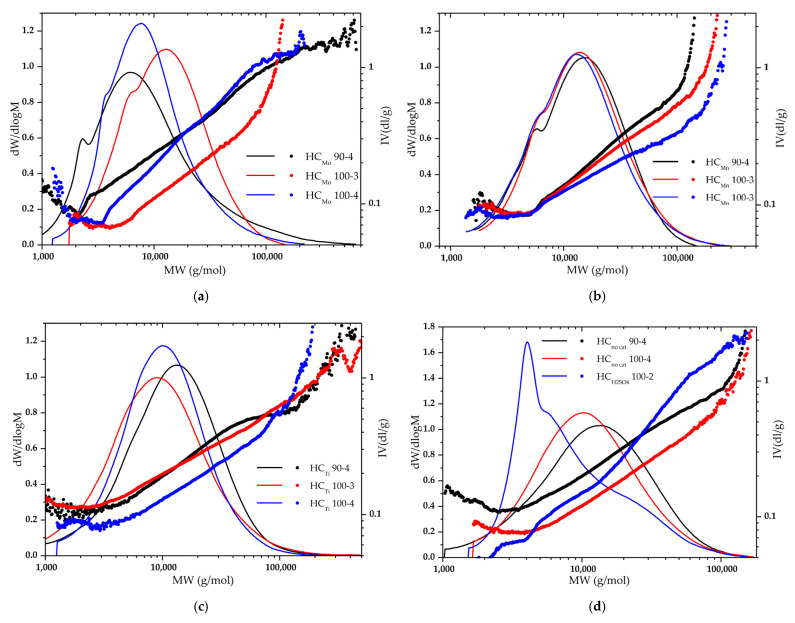
Molecular weight distribution curves and Mark–Houwink– Sakurada plots for the hemicelluloses obtained by oxidative delignification of spruce wood with the (**a**) (NH_4_)_6_Mo_7_O_24_, (**b**) MnSO_4_, (**c**) TiO_2_, and (**d**) H_2_SO_4_ catalysts and without them.

The molecular weight distribution (MWD) curves and MHS plots for the HCs obtained by oxidative delignification of spruce wood are presented in Figure 2 and the corresponding data are given in Table 2. 

According to the molecular weight distributions, all the HC samples are homogeneous with a slight polydispersity (1.8–3.3).

When the (NH_4_)_6_Mo_7_O_24_ catalyst is used at process parameters of 90 °C and 4 h, the obtained HC sample has a polydispersity index (PDI) of 3.354, which is explained by the presence of low molar mass fractions. The coefficient α = 0.45 suggests that the polysaccharide has a form of a linear random coil. As the process temperature increases to 100 °C, first (for 3 h) the HC molar mass slightly increase, the α coefficient grows to 0.63, and the MHS curve shifts (Figure 2a). This is indicative of a change in the HC conformation and the increased branching of the polymer, which is probably related to the partial oxidation of hydroxyl groups to carboxyl ones. With an increase in the processing time, partial hydrolysis of the side chains occurs, which can be concluded from the shift of the molecular weight distribution curve to the low molar mass region and an increase in the coefficient K (the weakened branching of polymer chains).

The HCs isolated in the presence of MnSO_4_ under varying conditions are fairly homogeneous: the MWD curves (Figure 2b) are almost identical, M_w_ ~ 19 kg/mol, and PD ~ 2. The main change is observed in the slope of the MHS curve (from 0.61 to 0.42), which can be attributed to the change in the polysaccharide chain conformation from the linear structure to a coil.

When TiO_2_ is used as a catalyst in oxidative delignification at 90 °C for 4 h, the obtained HC sample has a high polydispersity index (3.255) and a fairly high molar mass (M_w_ ~ 43 kg/mol). An increase in the process temperature to 100 °C leads to a shift of the MWD curve to the low molar mass region (M_w_ ~ 30 kg/mol) (Figure 2c). An increase in the process time significantly affects the polymer branching: the MHS curve shifts. This is, most likely, due to the partial oxidation of the HC hydroxyl groups, which leads to an increase in the polymer molar mass (M_w_ ~ 35 kg/mol) and in branching.

The HC samples obtained at a temperature of 90 °C for a process time of 4 h without a catalyst have a high molar mass (M_w_ ~ 47 kg/mol) (Figure 2d). This may be due to the mild conditions for the polysaccharide isolation when the depolymerization processes almost do not occur. However, the temperature growth intensifies the depolymerization processes, which is reflected in a decrease in the molar mass to M_w_ ~ 40 kg/mol. In this case, hydroxyl groups are partially oxidized, probably on the surface of a polysaccharide coil. This process is confirmed by a shift of the MHS curve and a decrease in the coefficient α to 0.41.

The presence of sulfuric acid as a catalyst in the reaction mixture does not allow one to obtain a significant HC yield. For instance, the product obtained at a temperature of 100 °C for 2 h has a low molar mass (M_w_ ~ 12 kg/mol). Analysis of the MWD curve profile (Figure 2d) reveals a small high molar mass region corresponding to the native HC and two peaks with M_w_ ~ 4 and ~6 kg/mol corresponding to the oligomeric products of HC hydrolysis, which is indicative of the inhomogeneous product composition.

### 2.4. Fourier-Transform Infra-Red Spectroscopy Study of the Hemicelluloses

**Figure 3:**
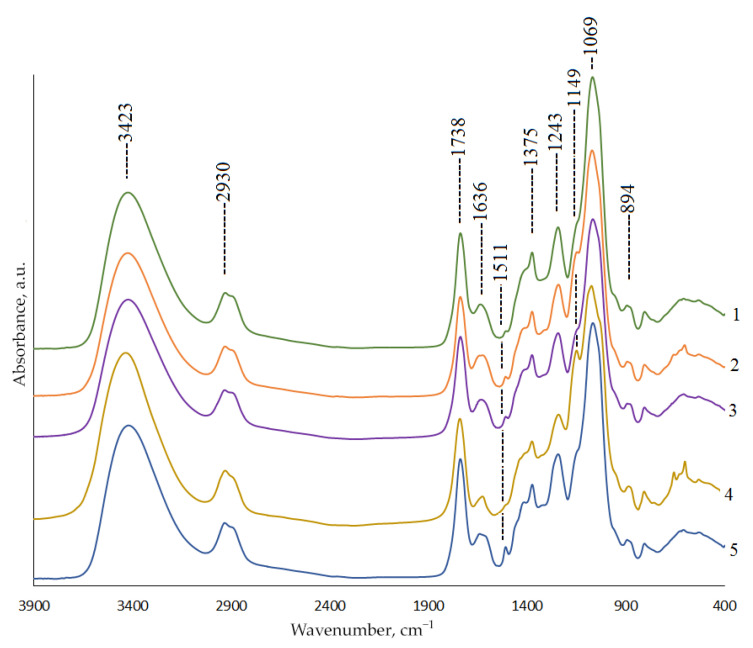
IR spectra of the hemicelluloses obtained by the oxidative delignification of spruce wood with (1) the (NH_4_)_6_Mo_7_O_24_, (2) MnSO_4_, (3) TiO_2_, and (4) H_2_SO_4_ catalysts and (5) without them.

The infra-red (IR) spectra of the HC samples include all the bands typical of heteropolysaccharides (Figure 3). Specifically, the characteristic high-intensity band of a hydroxyl group is observed around ~3423 cm^−1^, which points out strong intra- and intermolecular interactions between the polysaccharide chains [27].

The band around 2930 cm^−1^ was attributed to the C–H stretching vibration. The intense absorption band at 1069 cm^−1^ was attributed to the C–O–C and C–O–H stretching vibrations of a pyranose ring [1,27]. In addition, all the HC samples are acetylated, which is reflected in three acetyl ether bands at 1738 cm^−1^ (ester C=O), 1375 cm^−1^ (–C–CH_3_), and 1243 cm^−1^ (–C–O–). Furthermore, the characteristic absorption around 894 cm^−1^ corresponds to the typical signal of the β configuration in polysaccharides. The residual lignin absorption band at 1511 cm^−1^ is most pronounced in the spectrum of the HCs obtained without a catalyst, which evidences incomplete delignification. When the (NH_4_)_6_Mo_7_O_24_, MnSO_4_, and TiO_2_ catalysts are used, this band is insignificant [1]; in the case of the H_2_SO_4_ catalyst used, it is completely absent. -

### 2.5. Thermogravimetric Analysis of the Hemicelluloses

**Figure 4:**
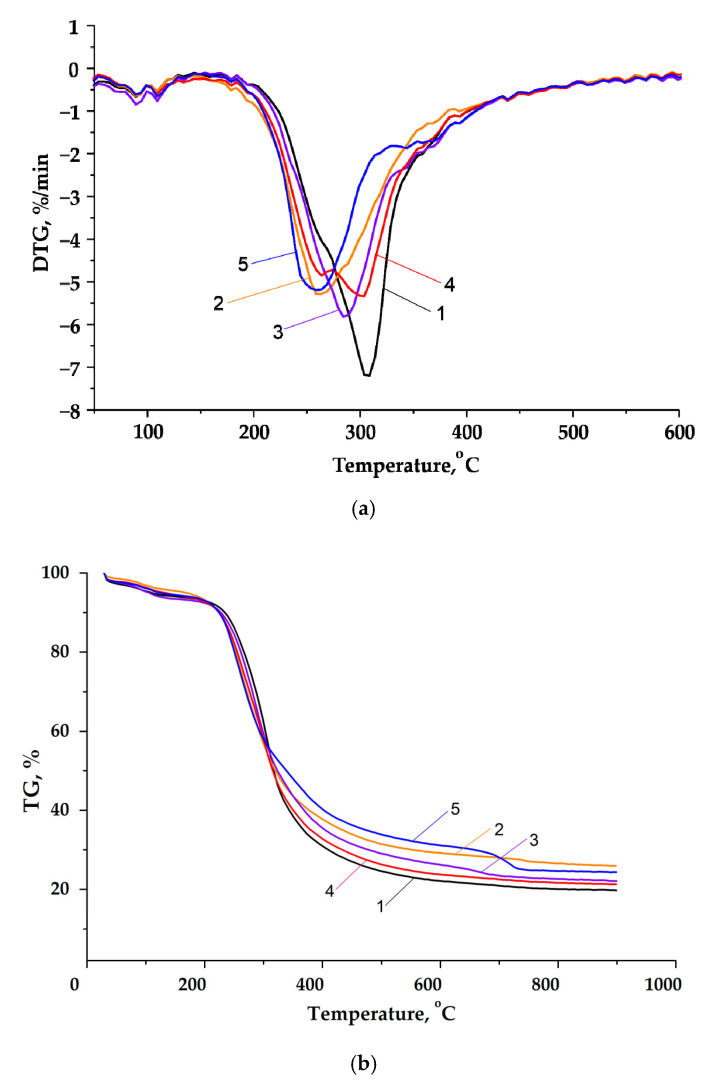
(**a**) DTG and (**b**) TG curves for the hemicelluloses extracted from the liquid products of the catalytic and noncatalytic delignification of spruce wood: (1) HC_w/o cat_, (2) HC_Mo_, (3) HC_Mn_, (4) HC_Ti_, and (5) ${\mathrm{HC}}_{H_{2}{\mathrm{SO}}_{4}}$.

The thermal properties of the HC samples were studied by the TGA. Figure 4a,b presents the derivative thermogravimetric (DTG) and thermogravimetric (TG) curves for the HC samples. 

The HCs isolated from spruce wood are resistant to heating to temperatures of up to ~100 °C and, upon further heating, start destructing at an increasing rate, as was shown by us in [1,31]. The thermal decomposition of the HC samples can be divided into three stages. At the first stage, the weight loss observed at temperatures of up to 100 °C is caused by the evaporation of adsorbed water. At the second stage, at relatively low (up to 200 °C) temperatures, the hydrolysis of ester groups apparently dominates and, due to the formation of acetic acid, the reactions of hydrolytic destruction of polysaccharides occur; further, the HCs are intensively decomposed, which begins at ~250 °C in all the samples.

As the temperature increases above 250 °C, the thermal destruction reactions with the hemolytic opening of the glycosidic and C–C bonds in the monosaccharide links already occur. In the investigated HC samples, the most rapid weight loss was observed at 270–320 °C. The intense decomposition ends at a temperature of ~340 °C for the four samples and at 300 °C for the sample obtained with the H_2_SO_4_ catalyst.

### 2.6. Elemental Analysis of the Hemicelluloses

The elemental composition of the HCs isolated from the liquid products of the catalytic and noncatalytic delignification of spruce wood in the acetic acid-hydrogen peroxide-water medium is given in Table 3. 

The elemental analysis revealed an increase in the oxygen fraction in the HC_Mo_ sample, which may be indicative of the presence of oxidized forms (uronic acids) in the structure. In the HC_Mn_ sample, the percentage of oxygen is higher than in the other samples: it approaches the values calculated for polyuronic acids. The increased number of carboxyl groups is consistent with the obtained data on antioxidant activity.

The elemental composition of the HC_Ti_ sample is similar to that of the sample obtained by the noncatalytic delignification; however, in the presence of the TiO_2_ catalyst, the HC partially oxidizes with the preserved native polymer structure, which is consistent with the GPC and FTIR data.

The maximum percentage of oxygen was found in the sample obtained using sulfuric acid, which is explained by the break of glycosidic bonds and further oxidation of the end groups.

### 2.7. Antioxidant Activity of the Hemicelluloses

**Figure 5:**
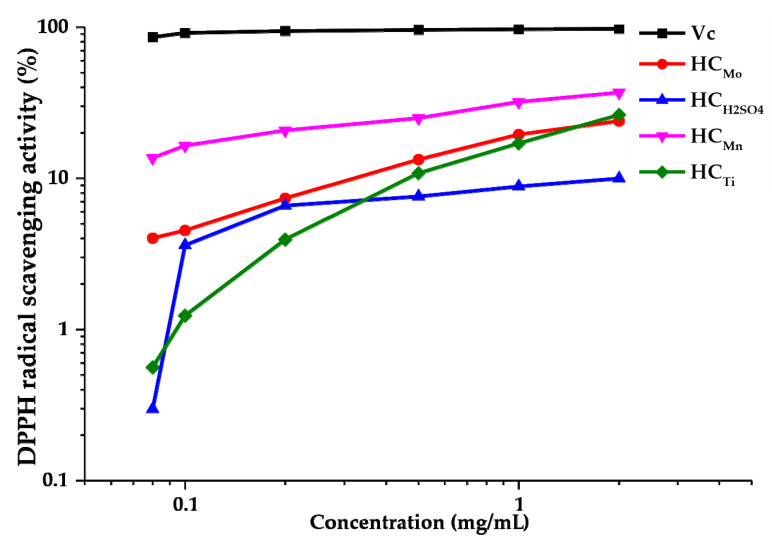
The activity of Vc and HC_Mo_, HC_Mn_, HC_Ti_, and ${\mathrm{HC}}_{H_{2}{\mathrm{SO}}_{4}}$ at different concentrations relative to the DPPH radicals.

A method widely used for determining the antioxidant activity is the 2,2-diphenyl-1-picrylhydrazyl (DPPH) free radical scavenging [32]. The stable nitrogen-centered lipophilic free radical DPPH exhibits the characteristic absorption at 517 nm and has a purple color in primary alcohols (methanol, ethanol and others) [32,33,34,35]. At the reduction by antioxidants to the nonradical DPPH-H form, the purple color rapidly disappears. The absorption capacities of the HCs were determined using the DPPH radical; the results are shown in Figure 5 in comparison with the Vitamin C (Vc) used for a positive control [32].

It is well-known that the specific functional groups, e.g., sulfate, amino, hydroxyl, and carboxyl ones, can be related to the antioxidative effect of polysaccharides [36]. It was found that all the HC samples have the lower activity with respect to DPPH as compared with Vc. All the HCs extracted with the catalysts showed the weakest scavenging effect at 0.08 mg/mL in the range of 0.29% for ${\mathrm{HC}}_{H_{2}{\mathrm{SO}}_{4}}$ to 13.66% for HC_Mn_. It is noteworthy that HC_Mn_ exhibited the strongest scavenging activity, but HC_H2SO4_ had the weakest scavenging activity over the entire range of 0.08–2.0 mg/mL. This is due to the fact that the contents of carboxyl and methoxyl groups of uronic acids, as well as the high content of hydroxyl groups positively affect the antioxidant activity of polysaccharides [36,37,38]. In addition, it was reported [39] that the galactose-rich polysaccharides have a relatively high antioxidant activity, which is consistent with our data on the monosaccharide composition. The comparable effect on the antioxidant activity is made by the polysaccharides contained in the HCs. The dependence of the antioxidant activity on the percentage of arabinoxylan, galactomannan, and galactoglucomannan in the HC structure was noticed in different studies [40,41,42].

## 3. Materials and Methods

### 3.1. Materials

The raw material used was sawdust (a fraction of 2.0–5.0 mm) of spruce (*Picea abies*) grown in the Krasnoyarsk Territory. The sawdust chemical composition was determined using conventional wood chemistry analytical methods [43]. The main spruce wood components were cellulose (44.4 wt%), lignin (30.6 wt%), hemicelluloses (22.6 wt%), extractive substances (1.8 wt%), and ash (0.6 wt%).

### 3.2. Spruce Wood Delignification and Extraction of the Hemicelluloses

**Figure 6:**
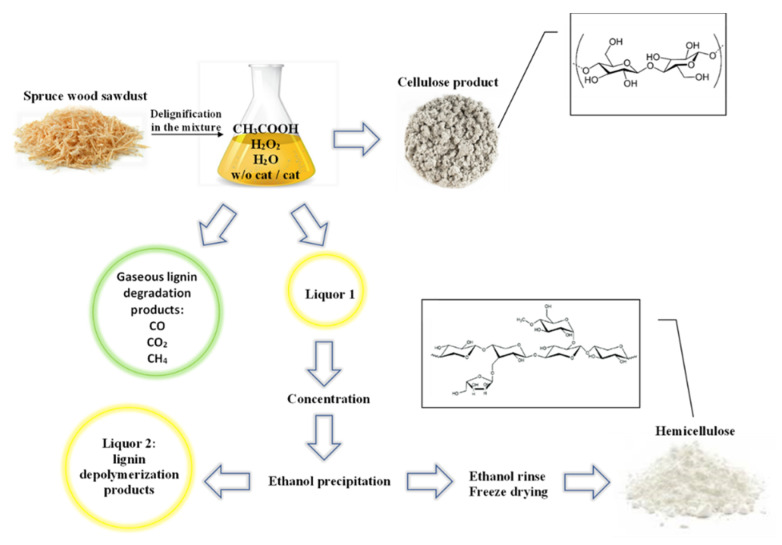
Scheme of the oxidative catalytic fractionation of spruce wood in the hydrogen peroxide-acetic acid-water medium with the formation of cellulose, hemicelluloses, and lignin products.

Figure 6 shows a scheme of the catalytic oxidative fractionation of spruce wood in the hydrogen peroxide-acetic acid-water medium with the formation of cellulose, hemicelluloses, and lignin products.

The shredded spruce wood was delignified in a 250-mL glass reactor equipped with a stirrer and a reflux condenser. The delignification solution consisted of glacial acetic acid (30 wt%), hydrogen peroxide (6 wt%), and distilled water. The LWR was 15. In this study, four catalysts (NH_4_)_6_Mo_7_O_24_, MnSO_4_, TiO_2_, and H_2_SO_4_ with the high delignification efficiency in a fraction of 1% of the wood weight were used. The delignification was performed at temperatures of 90 °C and 100 °C for 3 and 4 h under constant stirring. After the completion of the delignification process, a fixed residue (the cellulosic product) was separated from the delignification liquids by filtration on a Büchner funnel, washing until neutral pH, and drying to the air-dry state.

Liquor 1 (Figure 6) containing delignification liquids was concentrated on a rotary evaporator. Then, hot (~60 °C) water was added in a volumetric ratio of 1:1 and driven off in a rotary evaporator. This procedure was performed twice to remove acetic acid. The HCs were precipitated with a fivefold volume of ethanol (96 wt%) under slow stirring and then held at a temperature of 4 °C for 12 h. The obtained white curdled HC precipitate was separated from the lignin depolymerization products (liquor 2 in Figure 6) by filtration on a Büchner funnel, washed with ethanol, frozen, and dried in an Iney-6 freeze dryer.

The obtained HCs were analyzed by FTIR spectroscopy, GC, GPC, and TGA. The antioxidant activity was studied using compounds modeling free radicals: DPPH and hydroxyl radicals (ferrous sulfate FeSO_4_). The elemental composition was examined.

### 3.3. Monosaccharide Composition of the Hemicelluloses

To determine the monosaccharide composition, the HCs were hydrolyzed by a 2% HCl solution for 3 h. The individual composition and monosaccharide content in the hydrolysates were determined on a VARIAN-450 GC gas chromatograph equipped with a flame ionization detector on a VF-624ms capillary column with a length of 30 m and an inner diameter of 0.32 mm. The hydrolysate sample was pre-derivatized by the technique described in [44] with the formation of trimethylsilyl derivatives. The silylation reagent was a mixture of trimethylchlorosilane and hexamethyldisilazane in pyridine; sorbitol was used as an internal standard (IS). Standards for analyzing the hydrolysates were glucose, arabinose, galactose, sorbitol, mannose, and xylose (Panreac, Darmstadt, Germany).

### 3.4. Gel Permeation Chromatography

The weight average molar mass M_w_, number average molar mass M_n_, polydispersity index PDI, and K of the HC samples were determined by GPC using an Agilent 1260 Infinity II multi-detector GPC/SEC system with a refractive detector. The separation was made on two Agilent PL aquagel-OH columns using the solution of 0.1 M LiNO_3_ in water as a mobile phase. The column was calibrated using Agilent polyethylene glycol standards (US). The eluent flow rate was 1 mL/min and the sample volume was 100 μL. Before the analysis, the samples were dissolved in the mobile phase (~5 mg/mL) and filtered through a 0.45-μm Agilent PES membrane filter (Millipore, Burlington, MA, USA). The data collection and processing were performed using the Agilent GPC/SEC MDS software.

### 3.5. Fourier-Transform Infra-Red Spectroscopy Study

The IR spectra were recorded on a Shimadzu IRTracer-100 FTIR spectrometer (Japan). Specimens for recording the IR absorption spectra were pressed in tablets containing 3 mg of the sample in a potassium bromide matrix. 

### 3.6. Thermogravimetric Analysis

The thermogravimetric analysis was made on a NETZSCH STA 449 F1 Jupiter simultaneous thermal analysis instrument (Selb, Germany). The HC samples were analyzed in argon at a heating rate of 10 °C·min^−1^, temperatures from 30 to 900 °C, and protective and blowout gas flow rates of 20 and 50 mL∙min^−1^, respectively. The Al_2_O_3_ cylindrical crucible with a perforated cover was used and a reference was the empty corundum crucible with a cover. The instrument was calibrated according to the specification using reference substances supplied with the instrument. The sample weight for the analysis was determined on a Sartorius BP121S analytical lab scale digital balance. The measurement data were processed using the NETZSCH. Proteus Thermal Analysis.5.1.0 software supplied with the instrument.

### 3.7. Elemental Analysis

The elemental analysis of the HCs isolated from the liquid products of the catalytic and noncatalytic delignification of spruce wood in the acetic acid-hydrogen peroxide-water medium was made on a Vario El Cube ELEMENTAR CHNSO analyzer (Hanau, Germany).

### 3.8. Antioxidant Activity

The absorption capacity of DPPH was used to establish the HC antioxidant activity determined by the method described in [45] with some modifications.

A solution of DPPH in ethanol (0.2 mmol/L) was prepared before the UV measurements. The HC samples were dissolved in distilled water at a concentration of 0.08–2 mg/mL. The polysaccharide solutions (1 mL) were thoroughly mixed with 2 mL of freshly prepared DPPH and 2 mL of ethanol. The mixtures were well-mixed and kept at room temperature for 30 min in the dark. After that, the absorbance was measured at 517 nm against a blank. The lower absorption of the reaction mixture indicates the higher free radical scavenging activity, as can be seen in the inhibition percent versus compound concentration plot. In this study, Vc was used as a positive control. The experiments were repeated three times and the obtained values were averaged.

The DPPH scavenging ability was calculated using the equation:

(1) $$\mathrm{DPPH}\;\mathrm{Radical}\;\mathrm{Scavenging}\;\mathrm{Ability}\;(\%)=\left(1-\frac{A_{S}-A_{B}}{A_{C}}\right)\times 100\%$$

where A_C_ was the absorbance of the DPPH solution without a sample, A_S_ was the absorbance of the test sample mixed with the DPPH solution, and A_B_ was the absorbance of the sample without DPPH solution.

## 4. Conclusions

The oxidative catalytic and noncatalytic delignification of spruce wood in the acetic acid-water-hydrogen peroxide medium was studied. The investigated processes can be used to fractionate wood into two high-demand products: cellulose and hemicelluloses. The maximum hemicellulose yield (up to 10.9 wt%) was attained with the (NH_4_)_6_Mo_7_O_24_ and MnSO_4_ catalysts at a process temperature of 100 °C.

The isolated hemicelluloses consist mainly of galactoglucomannan and some amount of arabinoglucuronoxylan and are free of residual lignin. It was found that the spruce hemicelluloses isolated in the presence of the (NH_4_)_6_Mo_7_O_24_, MnSO_4_, and TiO_2_ catalysts have a narrow molecular weight distribution and a weight average molar mass of M_w_ = 16–42 kg/mol. The conformation and brunching of hemicellulose molecules are significantly affected by the process conditions and a catalyst used. The use of the water-soluble (NH_4_)_6_Mo_7_O_24_ and MnSO_4_ catalysts leads to the partial depolymerization intensified with increasing temperature, which is reflected in the change in the conformation and branching of polysaccharides. When the heterogeneous TiO_2_ catalyst is used, the molecular weight distribution and polydispersity of the obtained hemicelluloses are comparable with the products of the noncatalytic process. Sulfuric acid significantly accelerates the acid hydrolysis of hemicelluloses; as a result, an inhomogeneous product in the form of a mixture of mono-, di-, and polysaccharides is obtained for only 2 h. 

The hemicelluloses isolated from spruce wood are resistant to heating to temperatures of up to 100 °C; the most rapid weight loss is observed at 270–320 °C. The intense decomposition ends at 340 °C and 300 °C for the sample obtained with the H_2_SO_4_ catalyst. 

The hemicelluloses extracted with the (NH_4_)_6_Mo_7_O_24_, MnSO_4_, and TiO_2_ catalysts showed a fairly high antioxidant activity in the range of 5–44% at a concentration of 0.08–2 mg/mL, except for the hemicelluloses obtained with the sulfuric acid catalyst, which exhibit the low antioxidant activity over the entire concentration range.

The obtained hemicelluloses can be used in biomedicine and the fabrication of multicomponent coatings and fillers for the food and pharmaceutical industries.

## Figures and Tables

**Table 1:** Hemicellulose yields from the soluble products of oxidative delignification of spruce wood with and without catalysts at process temperatures of 90 °C and 100 °C and process times of 3 and 4 h.

Samples ^1^	Hemicellulose Yield (wt%) ^2^			
	90 (°C)		100 (°C)	
	3 (h)	4 (h)	3 (h)	4 (h)
HC_no cat_^3^	8.6	10.1	10.4	11.7
HC_Mo_^4^	7.9	9.2	10.1	10.9
HC_Mn_^5^	5.9	8.2	10.7	9.05
HC_Ti_^6^	5.8	8.0	8.4	7.0
${\mathrm{HC}}_{H_{2}{\mathrm{SO}}_{4}}\;$ ^7^	0.9	1.1	2.3	1.5

^1^ The ratio between the delignifying solution components CH_3_COOH:H_2_O_2_:H_2_O is 6:30:15; the amount of catalyst is 1 wt% of the wood weight; ^2^ Relative to the initial air-dry spruce wood; ^3^ The hemicelluloses obtained by noncatalytic delignification; ^4^ The hemicelluloses obtained in the presence of the (NH_4_)_6_Mo_7_O_24_ catalyst; ^5^ The hemicelluloses obtained in the presence of the MnSO_4_ catalyst; ^6^ The hemicelluloses obtained in the presence of the TiO_2_ catalyst; ^7^ The hemicelluloses obtained in the presence of the H_2_SO_4_ catalyst.

**Table 2:** Molar mass characteristics of the spruce wood hemicelluloses.

HC Sample	Catalyst	T (°C)	t (h)	M_w_ (g/mol)	PDI	K	α
HC_Mo_ 90-4	(NH_4_)_6_Mo_7_O_24_	90	4	17,367	3.354	442.43	0.45
HC_Mo_ 100-3	(NH_4_)_6_Mo_7_O_24_	100	3	16,797	1.883	37.48	0.63
HC_Mo_ 100-4	(NH_4_)_6_Mo_7_O_24_	100	4	12,471	2.11	69.17	0.63
HC_Mn_ 90-4	MnSO_4_	90	4	18,963	2.022	56.36	0.61
HC_Mn_ 100-3	MnSO_4_	100	3	20,382	2.053	64.85	0.58
HC_Mn_ 100-4	MnSO_4_	100	4	19,061	2.198	272.03	0.42
HC_Ti_ 90-4	TiO_2_	90	4	42,793	3.255	472.17	0.33
HC_Ti_ 100-3	TiO_2_	100	3	29,962	2.612	128.00	0.46
HC_Ti_ 100-4	TiO_2_	100	4	35,363	2.646	273.17	0.35
HC_no cat_ 90-4	w/o cat	90	4	47,645	2.145	25.41	0.59
HC _no cat_ 100-4	w/o cat	100	4	40,885	2.917	148.32	0.41
${\mathrm{HC}}_{H_{2}{\mathrm{SO}}_{4}}$ 100-4	H_2_SO_4_	100	2	12,845	1.815	1.57	1.01

**Table 3:** Carbon, hydrogen, and oxygen contents in the hemicellulose samples.

Sample	C (%)	H (%)	O ^1^ (%)
HC_Mo_	40.96	5.96	53.08
HC_Mn_	39.74	5.92	54.34
HC_Ti_	42.10	6.08	51.82
${\mathrm{HC}}_{H_{2}{\mathrm{SO}}_{4}}$	36.44	5.77	57.78
HC_no cat_	42.85	6.14	51.01

^1^ Calculated as a difference 100% –C (%) –H (%).

## Data Availability

All the data generated during this study are included in this article.

## Sample Availability

Samples of the compounds are available from the authors.
